# Cryptocurrency Turmoil: Unraveling the Collapse of a Unified Stablecoin (USTC) through Twitter as a Passive Sensor

**DOI:** 10.3390/s24041270

**Published:** 2024-02-17

**Authors:** Stefano Ferretti, Marco Furini

**Affiliations:** 1 Department of Pure and Applied Sciences, University of Urbino Carlo Bo, 61029 Urbino, Italy; stefano.ferretti@uniurb.it; 2 Department of Communication and Economics, University of Modena and Reggio Emilia, 42100 Reggio Emilia, Italy

**Keywords:** stablecoin, cryptocurrency, opinion leader, Twitter conversations

## Abstract

This study sought to explore whether Twitter, as a passive sensor, could have foreseen the collapse of the Unified Stablecoin (USTC). In May 2022, in just a few days, the cryptocurrency went to near-zero valuation. Analyzing 244,312 tweets from 89,449 distinct accounts between April and June 2022, this study delved into the correlation between personal sentiments in tweets and the USTC market value, revealing a moderate correlation with polarity. While sentiment analysis has often been used to predict market prices, the results suggest the challenge of foreseeing sudden catastrophic events like the USTC collapse solely through sentiment analysis. The analysis uncovered unexpected global interest and noted positive sentiments during the collapse. Additionally, it identified events such as the launch of the new Terra blockchain (referred to as “Terra 2.0”) that triggered positive surges. Leveraging machine learning clustering techniques, this study also identified distinct user behaviors, providing valuable insights into influential figures in the cryptocurrency space. This comprehensive analysis marks an initial step toward understanding sudden and catastrophic phenomena in the cryptocurrency market.

## 1. Introduction

**Figure 1:**
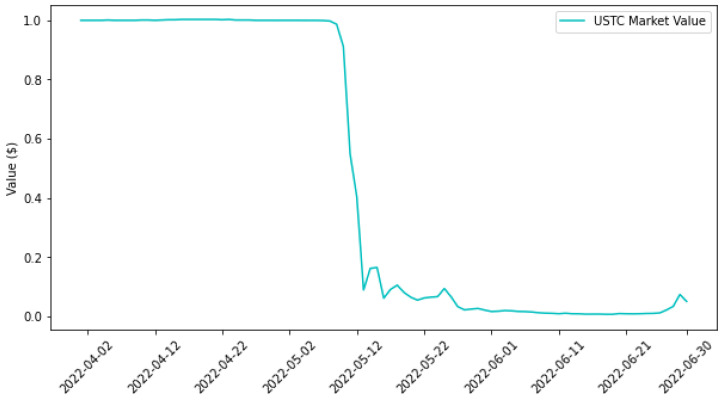
USTC trend from April to June 2022; source: Coin Market Cap.

The crypto world witnessed a significant event in May 2022 with the collapse of the Unified Stablecoin (USTC) ecosystem. USTC, a decentralized finance (DeFi) platform, aimed to establish a stablecoin pegged to the US dollar. Despite its well-thought-out design and the backing of a diverse cryptocurrency basket, the platform faced an unexpected downturn, initiating a precipitous decline on 7 May 2022 and culminating in a near-zero valuation on 11 May 2022, as illustrated in Figure 1 [1,2].

The USTC project, in essence, aimed to tackle a fundamental challenge in the cyber scenario: the creation of a stable cryptocurrency. The prospect of a cryptocurrency untethered from the volatility of exchange rates presented a compelling narrative, encompassing stability, widespread adoption, economic transactional efficiency, financial inclusivity, and a reduction in financial risks [3,4].

Stablecoins, constituting a distinctive category within the cryptocurrency realm, aspire to maintain a consistent value relative to an underlying asset, setting them apart from the typical volatility associated with cryptocurrencies [5]. In the midst of this, the Terra blockchain protocol emerged as a significant player, facilitating the creation of stablecoins pegged to fiat currencies. Terra introduced an algorithmic bank mechanism, dynamically adjusting the stablecoin supply based on the changing demand, thus ensuring sustained stability. Notably, Terra’s stablecoins, such as UST and KRT, gained popularity for their versatile applications in DeFi, payments, and secure value storage. The native cryptocurrency of Terra, LUNA, also assumed a pivotal role, acting as collateral for borrowing stablecoins. Terra’s DeFi applications, including Anchor and TerraSwap, further enriched the landscape by offering lending, decentralized exchange, and savings platforms. The unique approach of Terra to stablecoins and its seamless integration into DeFi applications signified a notable development in the evolving blockchain technology, capturing interest in the digital economy due to its features of anonymity and transparency [6].

This study focuses on the overarching theme of the collapse of the Terra stablecoin [7] and is motivated by the striking assertion from the Terra project’s co-founder Do Kwon, who predicted a high failure rate of companies entering the cryptocurrency market [7]. In essence, our research questions are the following: Can Twitter be used as a passive sensor to predict a cryptocurrency collapse? Is sentiment analysis a reliable predictor?

To address these questions, we focused on the USTC collapse and on the Twitter conversations during the months of April, May, and June 2022. It is worth noting that the collapse of the currency began on 7 May and ended four days later when its value became nearly zero. During the analyzed period, there were 244,312 tweets from 89,449 distinct accounts on the topic. Throughout the analysis, intriguing insights emerged. The geographical analysis uncovered unexpected global interest, with vibrant discussions extending beyond expected regions like the United States. Countries such as Brazil, India, Nigeria, Indonesia, and Bangladesh actively participated in the conversation. Further examinations, including sentiment analysis, hashtag studies, and polarity and subjectivity analyses, brought forth nuanced patterns and trends within the Twitter conversations. Of particular note was the discovery of distinct peaks in sentiment during the collapse, reflecting a mix of negative and positive sentiments. These data confirm that there was entertainment in watching companies die [7]. An intriguing positive surge on 28 May aligned with the launch of the new Terra blockchain, now currently referred to as “Terra 2.0”, adding an extra layer of complexity to the sentiment landscape. Going beyond sentiment, during polarity and subjectivity analyses, we found a moderate correlation between polarity sentiments and the USTC market value. To identify opinion leaders, the follower count proved to be inadequate. Turning to more sophisticated machine learning clustering techniques, the study successfully discerned distinct user behaviors, effectively singling out individuals deemed opinion leaders.

In conclusion, this multifaceted analysis serves as a beacon, illuminating diverse perspectives, intricate sentiments, and influential figures within the cryptocurrency space and offering valuable insights into the collapse of the Terra stablecoin.

The remainder of this paper is organized as follows. In Section 2, we review studies in the field of stablecoins and social media; Section 3 shows the idea of this study, whereas Section 4 shows the experimental setting and the obtained results. A discussion, limitations, and future work are presented in Section 5, and conclusions are drawn in Section 6.

## 2. Related Work

Twitter has served as a crucial source of information across various domains, playing a pivotal role in activities ranging from identifying opinion leaders and conducting human behavior studies to crisis management and information security. Regrettably, the recent change in Twitter’s leadership, marked by its rebranding as X, has led to the unavailability of several APIs, including Academic APIs, thereby hindering access to valuable data. Nevertheless, despite this setback, in the recent past, Twitter has been extensively employed in scholarly works. In the subsequent discussion, we briefly delve into some of the latest methodologies employed in utilizing Twitter as a passive sensor, particularly in the realms of opinion leader identification and the application of sentiment analysis for scrutinizing cryptocurrencies.

### 2.1. Twitter as Passive Sensor

Twitter has been used as a passive sensor to capture diverse human activities and events, enabling researchers to delve into a broad spectrum of social phenomena, from disease outbreaks to political occurrences. Its expansive user base and real-time dynamics render it an optimal data source for the examination of human behavior and opinions. In the subsequent sections, we provide a concise overview of studies that leverage Twitter as a passive sensor across various scenarios.

In the realm of opinion leader analysis [8,9,10], diverse methodologies have been proposed to identify opinion leaders. Typically, these approaches involve a blend of visibility and community engagement metrics, encompassing factors like the number of followers, followings, likes, replies, quotes, and mentions. Some studies hinge on the follower count or post frequency, while others introduce advanced metrics integrating and weighting different parameters. The insights garnered from these studies offer substantial advantages for individuals engaged in social media communication strategies, spanning political campaigns, brand monitoring, and policymaking.

Several studies examining human behavior [11,12,13] have underscored the cost-effective-ness and non-intrusive nature of data collection through Twitter. These investigations collectively demonstrate the feasibility of passively acquiring social media data on a large scale. The practical implications span diverse scenarios, encompassing workplace performance, innovation diffusion, human mobility, and pandemic spread.

Within the domain of crisis management [14,15,16,17], various studies have concentrated on the identification and localization of events such as earthquakes, hurricanes, disease outbreaks, and wildfires. The outcomes underscore that Twitter feeds serve as a hybrid sensor system, highlighting the potential of social media content in monitoring events. These findings support the notion that individuals, functioning as sensors, offer timely and comparable results, thereby enhancing situational awareness and optimizing responses to various crises.

In the realm of information security [18,19,20], Twitter has been utilized as a passive sensor to monitor cyber-attacks, investigate security issues, and aid in terrorist detection. Typically, methodologies in this domain leverage sentiment analysis, natural language processing, and machine learning as data sources to track social data that may indicate potential information security incidents.

### 2.2. Opinion Leader Identification/Analysis

Grasping the dynamics of opinion leaders in social media is pivotal to understanding information dissemination, influence, and trend-setting within digital communities. Research in this field focuses on the identification, characteristics, and impact of opinion leaders across platforms [21]. Generally, these individuals wield significant influence on attitudes, beliefs, and behaviors [22]. In the social media context, opinion leaders are perceived as field experts, and recognizing them is crucial for enhancing recommendation accuracy and the efficacy of social media campaigns [8].

Numerous studies have probed the role of opinion leaders in social media. Turcotte et al. [22] experimentally assessed the impact of news recommendations from social media opinion leaders on media trust and information seeking. The results showed enhanced trust and an inclination to follow more news from the recommended media outlet, especially when the sharer was perceived as an opinion leader. Li et al. [23] introduced a novel social recommendation method centered on opinion leaders, outperforming other methods in accuracy. Kim and Dennis [24] explored the link between social media use, opinion leadership, and political participation, finding that opinion leaders are more politically active and influential in expressing and shaping political views on social media compared to non-leaders.

### 2.3. Sentiment Analysis and Cryptocurrencies

Sentiment analysis plays an important role in decoding public perception and emotions surrounding cryptocurrencies. By analyzing social media, news, and online discussions, it gauges market sentiment, impacting trading decisions. Positive sentiment often drives price surges, while negative sentiment can trigger selloffs. This intersection of technology and behavioral analysis helps investors navigate the volatile landscape of cryptocurrency markets, enhancing decision-making strategies.

Primarily, literature studies have focused on predicting cryptocurrency prices and/or trends. Oikonomopoulos et al. [25] employed a sentiment analysis of news and social media data, utilizing machine learning algorithms to predict Ethereum and Polkadot prices based on over 3 million tweets. In contrast, Kolonin et al. [26] proposed a practical framework for causal analysis using financial metrics and sentiment analysis. Abraham et al. [27] found that tweet volume, not sentiment, predicts the price direction, offering advantages to cryptocurrency users. Kraaijeveld and De Smedt [28] explored Twitter sentiment’s predictive power for major cryptocurrencies, discovering its impact on Bitcoin, Bitcoin Cash, Litecoin, EOS, and TRON. Arti et al. [29] focused on Bitcoin and Litecoin price prediction using a multi-linear regression model. Wu et al. [30] examined economic policy uncertainty’s effect on cryptocurrency returns. Valencia et al. [31] employed machine learning and sentiment analysis to predict Bitcoin, Ethereum, Ripple, and Litecoin markets, with neural networks outperforming other models. Abubakr et al. [32] studied the predictive ability of Twitter Happiness Sentiment for six major cryptocurrencies, finding significant nonlinear relationships. Finally, Pant et al. [33] explored the direct or indirect influence of Twitter sentiment on Bitcoin’s overall market value, achieving notable accuracy in sentiment classification and price prediction using recurrent neural networks.

## 3. Our Study

The goal of our study is to analyze the collapse of the USTC currency to ascertain the potential for predicting future financial crises. Identifying predictive features in the realm of cryptocurrency could empower individuals to take preemptive measures in future crisis management. Indeed, as stated by the Terra project’s co-founder projections, USTC’s downfall might not be an isolated incident [7]. Leveraging Twitter as a passive sensor, our study seeks to proactively understand potential collapses by analyzing conversations on Twitter. Inspired by the literature suggesting that sentiment analysis can forecast cryptocurrency market values, we aim to validate this approach for extraordinary events like the USTC currency incident. Furthermore, we will explore the insights derived from hashtag analysis, geolocation, and the identification of opinion leaders.

The proposed study represents a first step in the realm of financial crisis prediction, particularly within the cryptocurrency market. By leveraging Twitter as a passive sensor, we introduce a novel methodology aimed at proactively understanding and potentially predicting future collapses. Unlike traditional financial data sources, which often rely on lagging indicators, our study harnesses the real-time nature of social media conversations to capture emerging trends and sentiments. Our approach not only offers a timely and dynamic perspective on market dynamics but also provides an opportunity to identify predictive features that may go unnoticed by conventional analytical methods. By incorporating sentiment analysis, hashtag analysis, geolocation data, and the identification of opinion leaders, our study presents a holistic and multidimensional framework for crisis detection and management.

### 3.1. Sentiment Analysis

Sentiment analysis, a facet of natural language processing, aims to discern and extract subjective information from textual data, unraveling the emotional tone within a string of words. When applied to Twitter, it becomes a powerful tool for gauging sentiments in tweets—categorizing them as positive, negative, or neutral [34,35]. Its applications span diverse domains, from predicting stock prices [36,37,38] and assessing cultural heritage scenarios [39,40] to monitoring societal health [41,42,43], tracking public opinion on specific topics [44,45,46], and fostering cross-media conversations [47,48,49].

However, conducting sentiment analysis on Twitter poses distinct challenges inherent to the platform. Tweets, characterized by brevity, harbor informal elements like slang and abbreviations, are highly context-dependent, and may incorporate emojis and emoticons. Overcoming these hurdles necessitates the deployment of specialized sentiment analysis models meticulously trained on expansive and diverse Twitter-specific datasets. While our focus lies in exploring sentiment within the Twitter-sphere, this paper confines itself to leveraging well-established libraries tailored for sentiment analysis on this platform, recognizing that delving into novel techniques extends beyond its current scope.

### 3.2. Hashtag Analysis

On Twitter, a hashtag is a metadata tag denoted by a word or phrase preceded by the “#” symbol. These tags categorize and organize tweets based on specific topics, themes, or keywords. When users incorporate a hashtag into their tweets, it transforms into a clickable link, guiding users to a feed of related tweets.

The analysis of hashtags within Twitter conversations holds significant importance for various reasons, such as content categorization, aiding in the comprehension of relevant topics or themes, fostering community engagement by unraveling dynamics within user groups, and monitoring public opinion to discern shifts in perception. In essence, hashtags function as a potent tool for structuring and comprehending Twitter conversations. Analyzing them provides a valuable perspective for researchers, businesses, and organizations, offering insights into the dynamics, trends, and sentiments prevalent within the Twitter ecosystem.

In the specific case addressed by this study, the analysis of hashtags can help determine whether the collapse of the currency has caused a shift in users’ perceptions of the topic.

### 3.3. Geolocalization

Geotagged tweets, equipped with location information, enable mapping and analysis based on their geographical origin. The significance of these tweets lies in their ability to offer profound insights into regional trends, preferences, and opinions. They serve as a valuable tool for identifying activity hotspots related to events or products, monitoring disease spread or natural disasters, and gauging public sentiment on political matters at the regional level. Businesses and organizations operating in specific regions benefit from these data, tailoring their strategies to local needs. In the context of this study, analyzing hashtags assists in discerning the countries expressing interest in the topic.

### 3.4. Opinion Leaders

Opinion leader analysis is crucial when analyzing Twitter conversations for several reasons: their views can reach a broad audience, and therefore, their identification helps identify individuals with the potential to shape public opinion; their influence provides insights into the emergence and propagation of trends within Twitter conversations; they play a role in shaping and influencing the dynamics of online communities; and they can significantly impact the narrative during significant events or crises. In essence, opinion leader analysis is a valuable aspect of understanding the social dynamics within Twitter conversations. It provides insights into the influencers who shape discussions, impact trends, and contribute to the overall fabric of the Twitter community.

The challenge in identifying opinion leaders within Twitter conversations stems from the absence of universally agreed-upon metrics in the literature. Indeed, various proposals exist, often contingent on the specific context under examination. In this study, we did not exploit literature proposals, but we designed specific machine learning techniques to discern opinion leaders within Twitter conversations focused on cryptocurrency.

## 4. Experimental Analysis

To address our research questions, we leveraged a dataset comprising tweets related to the topic in the month preceding the collapse, during the collapse, and in the month following the collapse. As an initial analysis, we delved into the geolocation data of the tweets to discern their geographical origin. This examination proved valuable in understanding the geographic distribution of these posts and potentially unraveling patterns linked to regional sentiments or trends.

Moving on to our second analysis, we explored the hashtags used to characterize the conversations. This investigation aids in determining whether there were shifts in conversational topics during the pre-collapse, collapse, and post-collapse phases. Understanding changes in hashtag usage provides insights into the evolving themes and interests within the Twitter discourse.

Concluding our analyses, we performed sentiment analysis to gauge user mood, sentiment polarization, and the subjectivity of messages. These findings serve as crucial inputs to explore potential correlations with the market value of the currency. Lastly, we embarked on identifying opinion leaders within these conversations, investigating whether the number of followers serves as an indicator of their influence on others’ opinions. This multifaceted approach aims to unravel the intricate dynamics surrounding the discussed topic during critical phases.

### 4.1. Dataset

The dataset was acquired through the Academic Twitter API, focusing on conversations related to the Terra collapse. We specifically filtered tweets posted during the critical period of the Terra collapse, spanning from 1 April 2022 to 30 June 2022. Tweets were required to contain one or more of the following hashtags and/or keywords: Blockchain, UST, TerraUSD, crypto, cryptocurrency, cryptocurrencyMarket, wluna, cryptoNews, stablecoin, Terra, cryptomarket, and Terra. As a result, we obtained a dataset comprising 244,312 tweets authored by 89,649 unique accounts.

### 4.2. Geo-Spatial Analysis

The initial analysis focused on the origin of tweets by examining the geo-field within each tweet. Within the dataset, 185,343 (75%) tweets contained geolocation information, encompassing a total of 207 different countries.

**Figure 2:**
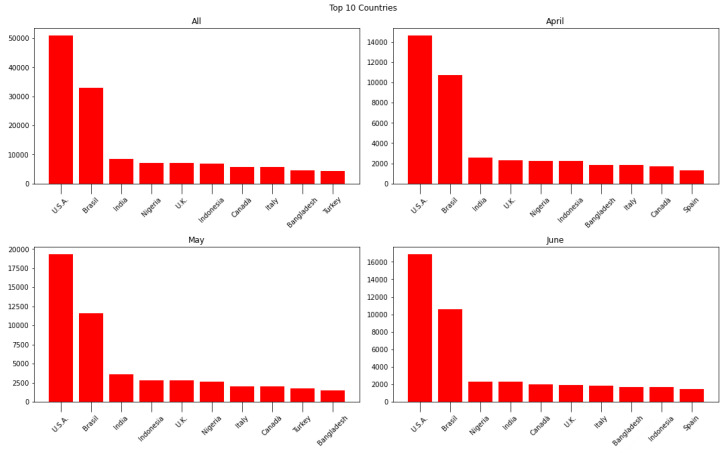
Geographical analysis: entire dataset (**top left**), April (**top right**), May (**bottom left**), and June (**bottom right**).

Figure 2 displays the top ten countries from which tweets were generated. At first glance, it might be surprising that South Korea is not listed among the top locations, given that the creators of Terra were based there. However, this can be explained by the fact that only a small percentage of people in South Korea use Twitter. Brazil stands out as one of the countries with the largest number of tweets. This was expected, considering that, at the time, Latin America ranked as the seventh-largest cryptocurrency market.

When reading this analysis, it is imperative to consider the population sizes of these nations. For instance, while India boasts a population of approximately 1.4 billion people, Canada’s population is significantly smaller, with around 38 million inhabitants. This vast discrepancy in population sizes can skew the interpretation of raw tweet counts, as countries with larger populations are expected to generate higher absolute numbers of tweets, regardless of the topic’s relevance or prevalence within that population. It is important to note that normalizing these results based solely on population figures is not feasible, as the normalization should ideally be performed relative to the total number of tweets posted on the platform, rather than the population size of each country. Unfortunately, without access to data on the overall volume of tweets from each country, such normalization remains unattainable. Consequently, while the geographical analysis provides insights into where the topic is being discussed, it is essential to refrain from drawing sociological inferences or conclusions based solely on the absolute number of tweets from each country. Instead, this analysis serves as a starting point for understanding the distribution of discussions on the topic, offering valuable context but requiring cautious interpretation.

### 4.3. Hashtag Analysis

Hashtag analysis is a powerful tool for researchers, marketers, and analysts seeking to extract meaningful insights from the vast and dynamic landscape of Twitter conversations. Analyzing hashtags in Twitter conversations is integral to extracting valuable insights and understanding the dynamics of online discussions. Hashtags serve as content categorizers, aiding in the organization of tweets around specific themes and topics. By identifying and analyzing popular hashtags, researchers can discern prevailing trends, community engagement levels, and key issues within the conversation. The usage and frequency of hashtags also facilitate real-time trend analysis, allowing researchers to stay informed about emerging topics.

**Figure 3:**
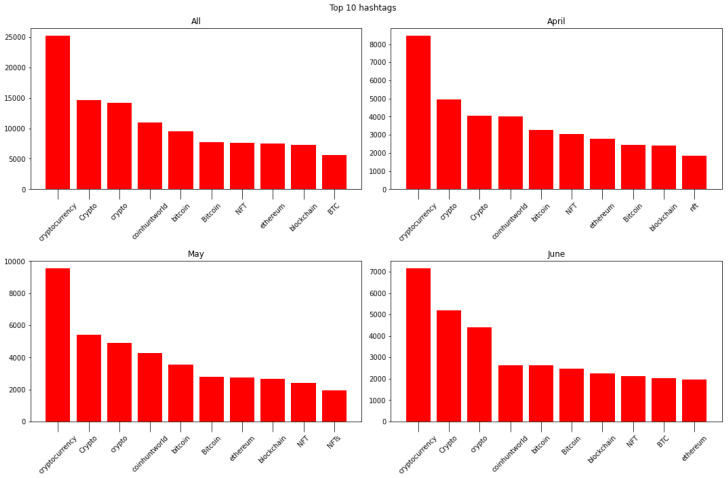
Hashtag analysis: entire dataset (**top left**), April (**top right**), May (**bottom left**), and June (**bottom right**).

In Figure 3, we present the top 10 hashtags utilized in conversations across various periods: the entire dataset and the individual months of April, May, and June. It is evident that the hashtags remain largely consistent, with some minor variations in their ranking. Additionally, the volume of tweets associated with these hashtags exhibits a comparable pattern, indicating a consistent usage trend over time. This observation underscores the persistence and stability of hashtag usage across the dataset, with only marginal fluctuations in their relative popularity.

### 4.4. Sentiment Analysis

The sentiment analysis was conducted using the well-established TextBlob library, enabling the calculation of sentiment, polarity, and subjectivity for each tweet. Upon mapping the entire dataset into three sentiment categories (positive, negative, and neutral), the results revealed 34% of tweets as positive, 8% as negative, and 58% as neutral.

**Figure 4:**
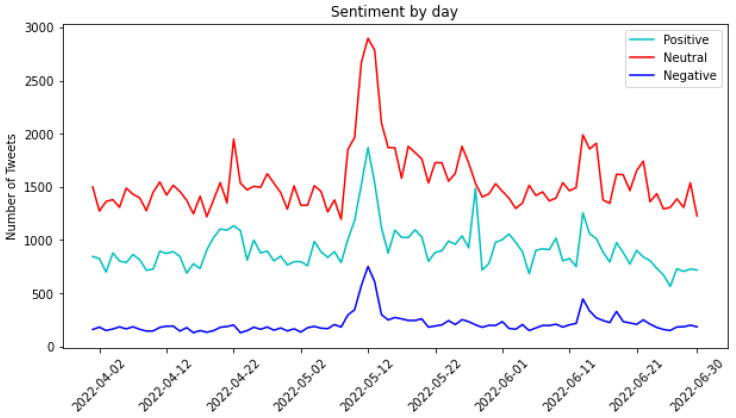
Classification of tweets according to their sentiment.

Figure 4 illustrates the sentiment classification of tweets grouped by day. In terms of absolute numbers, the count of neutral tweets consistently exceeded that of positive tweets, which, in turn, exceeded negative ones. On 8 May, the genuine collapse was triggered, reaching its climax on Black Wednesday, 12 May (indicated by spikes in the graph), when the entire Terra ecosystem faced significant challenges. Notably, on 12 May 2022, there was a surge in positive tweets. Surprisingly, individuals posted significantly more positive tweets than negative ones. This could be attributed to efforts to calm the market, preventing an impending crisis. Alternatively, there may have been excitement surrounding the collapse of the cryptocurrency, sparking surprise and curiosity.

Moreover, some instances revealed a sense of happiness or irony. This might be attributed to the notion that witnessing the downfall of entities can be perceived as a form of entertainment, as stated in [7]. Certain messages conveyed a positive tone, serving as advertisements to attract new followers and suggesting that following their profile could help avoid similar problems in the future. These messages aimed to present new opportunities to the audience. Simultaneously, other messages expressed satisfaction or relief at not having invested in cryptocurrency. Statements such as, “*the risks were always there, and I’m glad I didn’t take that risky bait*”, highlighted a sentiment of contentment.

Toward the end of May, there was another notable spike in positive tweets, possibly linked to the news that a new Terra blockchain was launched on 28 May, accompanied by its native token, $LUNA.

**Figure 5:**
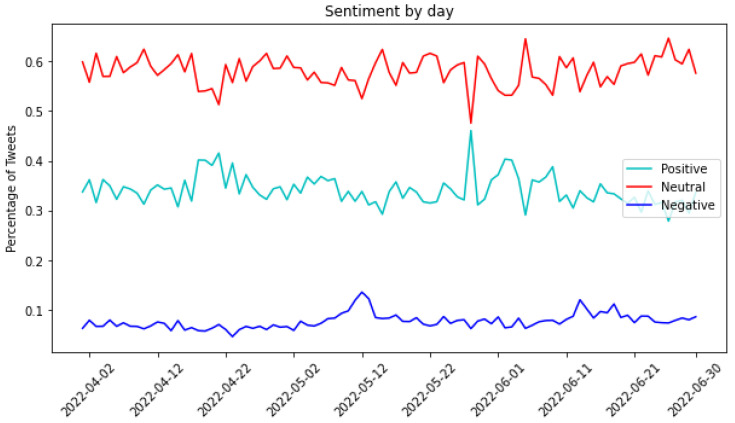
Percentage of tweets classified according to their sentiments.

Figure 5 provides an alternative perspective on the unfolding situation by illustrating the percentage distribution of tweets based on sentiment.

The negative sentiment line exhibits a notable spike commencing on 7 May and concluding on 13 May, aligning precisely with the critical days of the collapse, as previously discussed. Concurrently, there is a corresponding decline in the percentage of positive tweets during this period. Noteworthy is an additional spike on 28 May, coinciding with the launch of the new Terra blockchain. Interestingly, while the introduction of the new blockchain did not significantly impact the percentage of negative tweets, there appears to be a shift in the connotation of neutral tweets, transitioning toward a more positive sentiment. This is evident in the negative spike in the neutral line and the positive surge in the positive line during this specific time frame.

### 4.5. Polarity and Subjectivity

In TextBlob analysis, polarity and subjectivity are two key measures that provide insights into the sentiment and nature of a piece of text, such as a sentence or document. In summary, polarity gauges the sentiment expressed in a text, ranging from positive to negative, while subjectivity measures how subjective or opinionated the text is, ranging from objective to subjective. These measures are valuable in sentiment analysis and can provide a quantitative understanding of the emotional tone and nature of textual content. In particular, they are defined as follows:

- Polarity is used to measure the sentiment expressed in a piece of text and ranges from −1 to 1, where −1 represents a completely negative sentiment, 0 indicates a neutral sentiment, and 1 signifies a completely positive sentiment.
- Subjectivity is used to measure how subjective or opinionated a piece of text is. It ranges from 0 to 1, where 0 indicates a highly objective or factual statement, and 1 suggests a highly subjective or opinionated statement. A higher subjectivity score implies a more opinionated or subjective piece of text. A lower subjectivity score indicates a more objective or factual statement.

**Figure 6:**
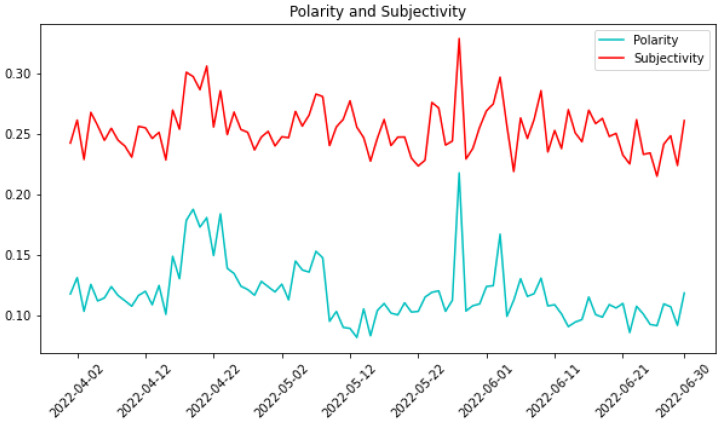
Polarity and subjectivity of tweets.

Figure 6 illustrates the dynamics of polarity and subjectivity values extracted from the analyzed tweets. The parallel trends observed in the two lines indicate that individuals tend to convey highly subjective content in their tweets. The most noteworthy spike occurred on 28 May, coinciding with the launch of the new Terra blockchain. This significant surge in both polarity and subjectivity values suggests a distinct shift in the sentiment expressed in tweets on that particular day. The heightened subjectivity implies that tweets during this period were notably opinionated, while the elevated polarity suggests a notable shift toward either positive or negative sentiments.

This nuanced analysis of polarity and subjectivity values provides valuable insights into the emotional tone and subjective nature of the Twitter conversations, particularly highlighting the pronounced impact of the launch of the new Terra blockchain on the sentiment expressed in tweets.

### 4.6. Market Value and Correlation with Tweet Sentiment

While people posted their tweets, the USTC Terra was out in the market. In the subsequent analysis, our goal is to discern whether a correlation existed between the market value of the USTC Terra and the sentiment of Twitter conversations. From 1 April to 30 June, a comprehensive analysis was conducted to compare daily market values with various tweet metrics, including absolute counts of positive, neutral, and negative tweets, as well as the total volume of tweets. The study further explored correlations with polarity and subjectivity measures derived from the tweet content. Additionally, correlations with the daily percentages of positive, negative, or neutral tweets were investigated.

This comprehensive analysis, detailed in Table 1, has revealed intriguing insights. The moderate correlation observed with polarity (0.41) suggests a discernible connection between the overall sentiment expressed in tweets and the underlying market dynamics. Furthermore, a noteworthy negative correlation of −0.43 with the percentage of negative tweets adds a layer of nuance to the relationship. Based on these numerical associations, there does not seem to be a clear indication that the sentiment of conversations could have forewarned the collapse that Terra underwent.

### 4.7. Is the Number of Followers Useful in Identifying Opinion Leaders?

The conventional method for pinpointing opinion leaders often involves associating them with the accounts boasting the highest follower counts. However, this approach is inherently flawed for two primary reasons: firstly, contemporary content algorithms on social media platforms prioritize discovery, granting smaller creators the potential for substantial reach and influence; secondly, the count of followers can be artificially inflated by fake accounts and bots, rendering it an unreliable metric [8]. Table 2 outlines the top 15 accounts within our dataset based on the follower count.

This renders “followers” an inadequate metric for identifying opinion leaders. For example, the top account, boasting over 61 million followers, posted merely two tweets, garnering a meager 158 likes, 8 quotes, and 54 replies, and a mere 46 accounts retweeted at least one of its two tweets. Evidently, this account falls significantly short of being considered an opinion leader on the given topic.

**Figure 7:**
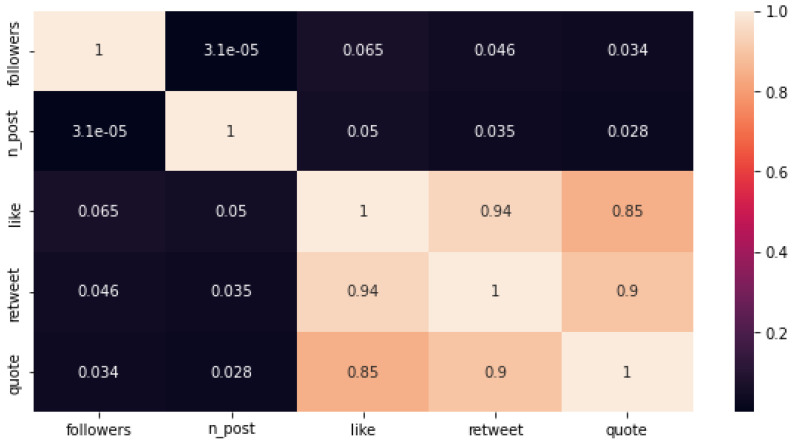
Correlation between the different metrics used to characterize Twitter users and posts.

Supporting the notion that opinion leaders and followers are not necessarily intertwined, Figure 7 illustrates the correlation among the key metrics characterizing Twitter users and their posts. A notable finding emerges concerning the correlation between the number of followers and interactions with posted tweets. Contrary to the assumption that a larger follower count invariably leads to more engagement, this is not always the case. Users with a modest following might experience greater interaction with their tweets than those with a substantial following. This underscores that the substance and relevance of the content play a pivotal role in driving engagement, surpassing the significance of the follower count alone.

Another noteworthy result highlights a robust correlation between likes, retweets, replies, and quotes. Likes serve as a straightforward indication of user appreciation, while retweets extend the tweet’s reach by allowing users to share it with their followers. Replies and quotes facilitate conversation around a tweet, either through direct responses or by adding user commentary. These diverse forms of engagement often synergize, with tweets accumulating likes, retweets, and replies simultaneously, contributing to the tweet’s overall virality and impact as it gains traction across the platform.

### 4.8. Opinion Leader Identification

To determine who the opinion leaders are, we used parameters that measure the degree of interaction with a tweet: likes, retweets, replies, and quotes. We calculated these parameters for each individual tweet, and then we calculated how many likes, retweets, replies, and quotes each account received during the analyzed period.

We used k-means, an unsupervised learning technique, to group these accounts based on their similarity. K-means is a popular clustering algorithm used in unsupervised machine learning to partition a given dataset into k clusters based on the similarity of their features. It has several advantages, including its simplicity, scalability, and efficiency on large datasets. The algorithm works by iteratively assigning data points to the nearest cluster centroid and updating the centroids based on the mean of the data points assigned to the cluster. The process continues until the centroids no longer move significantly, or a maximum number of iterations is reached.

The k-means algorithm requires the number of clusters (k) to be specified beforehand, which can be determined through techniques such as the elbow method or silhouette analysis. In this paper, we consider the silhouette score, a metric that measures how well each data point fits into its assigned cluster compared to other clusters. The silhouette score ranges from −1 to 1, with higher values indicating that a data point is well matched to its cluster, while poorly matched points have negative scores. To find the optimal value of k (the number of clusters) in k-means, we can compute the silhouette score for different values of k and choose the k value with the highest average silhouette score. This process involves fitting the k-means algorithm to the dataset multiple times, each time with a different value of k. For each value of k, we compute the silhouette score for each data point and take the average across all data points to obtain the average silhouette score for that value of k. The k value with the highest average silhouette score is considered to be the optimal value for clustering the data.

By applying the silhouette algorithm to our dataset, we obtained very similar scores for k values that range from 2 to 3.

Table 3 shows the results obtained with k = 3. This means that the clustering process produces three different classes. In the first cluster (the one very close to zero), there are 61,967 accounts that can be defined as accounts that were unable to influence the opinions of others. In the second cluster, there are two accounts that can be identified as opinion leaders, and in the third cluster (the one farthest from zero), there is one account that can be defined as a top opinion leader. As can be observed from Table 3, posting a high number of tweets does not guarantee becoming an opinion leader, just as having a large number of followers does not guarantee a high number of interactions.

## 5. Discussion

The results obtained reveal that predicting the collapse of a cryptocurrency is a complex task. While sentiment analysis techniques are effective in forecasting market values under normal circumstances, they do not distinctly signal an impending crisis. In our study, we identified only a moderate correlation, which cannot serve as a definitive alert. It serves as an indication that something is unfolding, yet it falls short of guaranteeing an imminent disaster. Nonetheless, this work represents an initial stride toward crisis management. Subsequently, we will discuss the possible generalization of our approach, and we will present the primary theoretical and practical implications stemming from our study.

### 5.1. Generalizability

In exploring the potential for the generalization of our approach, it is crucial to recognize the foundational principles and methodologies that underpin our analysis. By focusing on the dynamics of Twitter conversations surrounding a significant event like the USTC collapse, we have developed a framework that can be applied to a wide range of contexts and topics within the realm of social media analysis. Indeed, our methodology incorporates a multifaceted approach that includes sentiment analysis, hashtag studies, and user behavior analysis. These techniques are not limited to specific events but can be adapted and utilized to investigate diverse subjects across various industries and domains. Furthermore, our use of sophisticated machine learning clustering techniques to identify opinion leaders showcases the scalability and adaptability of our methodology.

### 5.2. Theoretical Contributions

The theoretical contributions of this study can enrich the academic understanding of cryptocurrency markets and predictive analytics in the following ways:

- Limits of sentiment analysis: This research highlights the limitations of using sentiment analysis as a predictive tool in the context of cryptocurrency market anomalies. This theoretical contribution challenges existing assumptions and theories that suggest sentiment analysis as a reliable indicator of market movements [33], paving the way for a more nuanced understanding of its applicability and boundaries. Additionally, it is worth noting that other studies also acknowledge the challenges of using sentiment analysis to predict cryptocurrency prices [27].
- Cryptocurrency market anomaly theory: This study contributes to the development of a theoretical framework specific to cryptocurrency market anomalies. This may involve rethinking existing theories or developing entirely new paradigms that explain the sudden and unpredictable nature of events such as market collapses in the cryptocurrency domain. Several studies have demonstrated the feasibility of using sentiment analysis to anticipate cryptocurrency market trends [33]. However, it is crucial to note that such analyses are commonly conducted in regular market contexts, whereas our study focuses on an exceptional scenario. Our data highlight that applying this type of analysis would not have provided useful predictions for the sudden collapse of the currency. This does not contradict previous research findings but further emphasizes the anomaly of a collapse that was so abrupt and unforeseen that it resulted in the loss of the entire value in just two days.
- Predictive model enhancements: This study sets the stage for theoretical advancements in predictive modeling for cryptocurrency markets. By identifying the limitations of current approaches, it prompts theoretical discussions on refining existing models or developing entirely new ones that can better capture the complexities of these markets, especially during critical events.

### 5.3. Practical Contributions

The practical contributions of this study can be significant in several aspects:

- Risk assessment and management: Understanding that sentiment analysis might not be effective in predicting anomalies, such as rapid collapses in cryptocurrency values, contributes to more informed risk assessments. This insight can be valuable for investors, financial institutions, and individuals involved in the cryptocurrency market seeking to adjust their risk management strategies.
- Research guidance: This study contributes to the academic community by highlighting the need for further research into alternative indicators and models for predicting market anomalies in the cryptocurrency space. This can guide future studies to explore innovative approaches that go beyond traditional sentiment analysis.
- Technological advancements: This study prompts discussions on the refinement and development of more sophisticated models and tools for analyzing cryptocurrency markets. This could stimulate technological advancements in the field of predictive analytics, offering improved methods for anticipating market behaviors and anomalies.

### 5.4. Limitations and Future Work

This study marks the initial phase of investigating the collapse of a cryptocurrency by employing sentiment analysis and opinion leader identification. Our methodology involved crucial decisions, such as selecting a sentiment analysis library and utilizing machine learning for opinion leader identification, relying on various Twitter metrics. While our approach treats all interactions equally, acknowledging that studies assign different weights to interactions is essential. Assigning varied weights considers the quality, relevance, and impact of each interaction, recognizing that a retweet may hold more significance than a simple like, indicating content deemed share-worthy. Similarly, replies or quotes may carry more weight if they spur conversations or debates, reflecting active engagement. Despite the challenge of determining optimal weights, incorporating nuanced interactions provides a more accurate measure of influence, identifying opinion leaders with subtle yet substantial impacts. Acknowledging potential limitations, this study suggests that future developments could explore refining interaction weights for a more context-specific understanding of opinion leadership.

## 6. Conclusions

This study utilized Twitter as a passive sensor to gauge public sentiments during the collapse of the Terra stablecoin project in April, May, and June 2022. Geographical analysis revealed significant engagement not only from the United States but also from unexpected countries, like Brazil, India, Nigeria, Indonesia, and Bangladesh. Text analysis, including hashtag studies, showcased consistent usage patterns irrespective of USTC market fluctuations. Sentiment analysis indicated peaks during the collapse, encompassing both negative and positive sentiments, with some accounts proposing alternative solutions. An intriguing positive surge on 28 May coincided with the launch of the new Terra blockchain algorithm. Polarity and subjectivity analyses underscored the subjective nature of tweet content, with opinions prevailing over factual information. Exploring correlations between personal sentiments and the USTC market value revealed a moderate correlation with polarity. Efforts to identify opinion leaders using follower counts were deemed inadequate, prompting the use of machine learning clustering techniques, which effectively discerned distinct user behaviors and identified influential figures. This comprehensive analysis sheds light on diverse perspectives and sentiments within the discourse on cryptocurrency issues.

In summary, the obtained results underscore the complexity involved in predicting the collapse of a cryptocurrency, emphasizing the need for extensive efforts to anticipate disastrous scenarios. Successfully foreseeing such events demands meticulous analysis and a comprehensive understanding of various factors affecting the market. Despite the challenges, this work marks an initial step toward the effective management of crises in the cryptocurrency landscape. It highlights the importance of ongoing research and the continuous refinement of predictive models to enhance the resilience of the financial ecosystem.

## Figures and Tables

**Table 1:** Correlation between the USTC market value and tweet sentiment.

	Correlation Value
Positive tweets	−0.105873
Neutral tweets	−0.213339
Negative tweets	−0.280721
Posted tweets	−0.199708
Tweet polarity	0.417790
Tweet subjectivity	0.171227
Percentage of positive tweets	0.228058
Percentage of neutral tweets	−0.009315
Percentage of negative tweets	−0.434704

**Table 2:** Top 15 opinion leader accounts according to the number of followers. The usernames have been partially obscured with *** for privacy reasons.

User	Followers	n_post	Likes	Retweets	Replies	Quotes
	(×1000)					
CN ***	61,082	2	158	46	54	8
Ma ***	34,853	1	7261	1359	468	445
WS ***	20,468	2	48	26	8	6
aa ***	19,647	2	180	18	14	4
Fo ***	18,704	5	561	198	181	94
nd ***	17,697	7	10,267	1669	711	487
FA ***	17,230	1	10,083	679	231	114
AB ***	13,245	2	123	26	33	6
uf ***	11,092	1	1000	157	51	14
Ti ***	10,323	2	49	8	2	2
bi ***	10,157	6	23,633	6525	14,949	1409
bu ***	9150	2	661	185	238	253
mc ***	8893	1	4	0	2	0
ht ***	8698	2	30	9	26	8
cz ***	8132	24	244,653	39,912	29,017	4896

**Table 3:** Accounts that emerge as opinion leaders with 3-means analysis. The usernames have been partially obscured with ** for privacy reasons.

Username	Followers	n_post	Likes	Retweets	Replies	Quotes
	(×1000)		(×1000)	(×1000)	(×1000)	(×1000)
Cr **	1530	69	600	556	189	377
Wa **	1708	78	301	54	38	8
Ai **	1456	60	307	309	63	58
ai **	1564	43	256	252	42	35
LB **	438	40	45	35	84	24
ku **	2375	24	50	19	94	4
Ai **	1564	13	126	114	31	56

## Data Availability

The data that support the findings of this study were collected through the Academic Twitter API.
